# Sterile Endocarditis and Splenic Infarct: A Rare Masquerading Presentation of Granulomatosis With Polyangiitis in a Patient With Pulmonary Aspergillosis

**DOI:** 10.7759/cureus.62190

**Published:** 2024-06-11

**Authors:** Visvarath Varadarajan, Viswanathan Pandurangan, Devasena Srinivasan, Leena Joseph, Arumugam Vasugi

**Affiliations:** 1 General Medicine, Sri Ramachandra Institute of Higher Education and Research, Chennai, IND; 2 Pathology, Sri Ramachandra Institute of Higher Education and Research, Chennai, IND

**Keywords:** small-vessel vasculitis, anti-neutrophil cytoplasmic antibody-associated vasculitis, pulmonary aspergillosis, splenic infarcts, marantic endocarditis, granulomatosis with polyangiitis (gpa)

## Abstract

Granulomatosis with polyangiitis (GPA) is a rare multisystem disease characterized by vasculitis affecting small vessels, resulting in the formation of necrotising granulomata, primarily affecting the lungs, the upper respiratory tract, and kidneys. Almost all patients have upper and lower respiratory involvement; up to 85% of patients with GPA develop kidney disease within two years of diagnosis. Cutaneous, neurological, and ocular manifestations are also seen with varying frequencies. However, cardiac manifestations of the disease are rare and scarcely reported in the literature. Here, we report a case of a 65-year-old female with an initial diagnosis of pulmonary aspergillosis based on the presence of septate hyphae branching at acute angles on lung biopsy and elevated serum galactomannan, who, over the following months, developed a multitude of issues such as myocardial infarction, sterile endocarditis, splenic infarction, and heart block, as well as the challenges faced in establishing a diagnosis and managing its complications.

## Introduction

Small-vessel vasculitides can broadly be categorised as immune-complex-mediated vasculitides, vasculitides secondary to infectious or inflammatory syndromes, and pauci-immune vasculitides, of which the latter group consists of the vasculitides characterised by the presence of anti-neutrophil cytoplasmic autoantibodies (ANCAs). Based on their immunofluorescence pattern, ANCAs are divided into perinuclear p-ANCA (which collects around the negatively charged nucleus) and cytoplasmic c-ANCA (which stains diffusely throughout the cytoplasm) [1].

Granulomatosis with polyangiitis (GPA) is an uncommon necrotising small-vessel vasculitis typically associated with c-ANCA. This disease notably involves the respiratory tract and the kidneys, often involving the eyes, skin, nerves, and peripheral vessels. Cardiac involvement is rare. The rarity of the disease, the multifariousness of its presentations, and the tendency of its clinical and investigational profile to mimic more common granulomatous inflammatory diseases can pose a diagnostic challenge. In this report, we present the case of a patient whose underlying GPA masqueraded with a tuberculosis-like picture initially and infective endocarditis later before being unmasked.

## Case presentation

**Figure 1:**
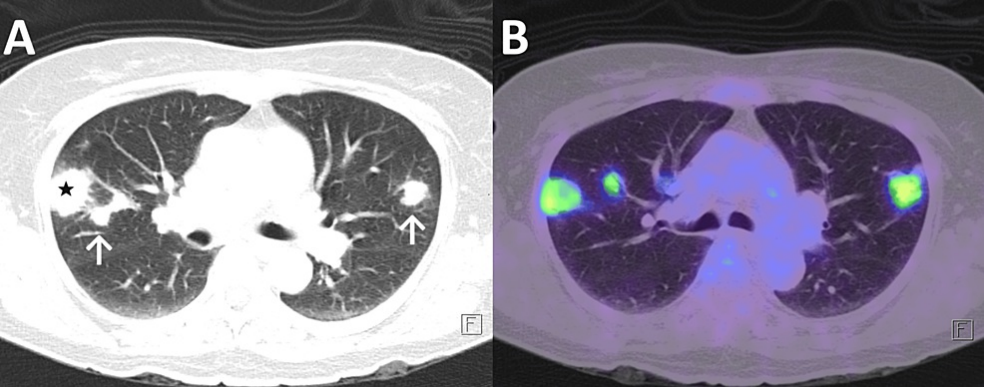
PET-CT. Pane A: Lung window showing right lung consolidating lesion (black star) with nodules in both lungs (white arrows). Pane B: PET window showing 18-fluorodeoxyglucose avidity in the lesion and nodules.

**Figure 2:**
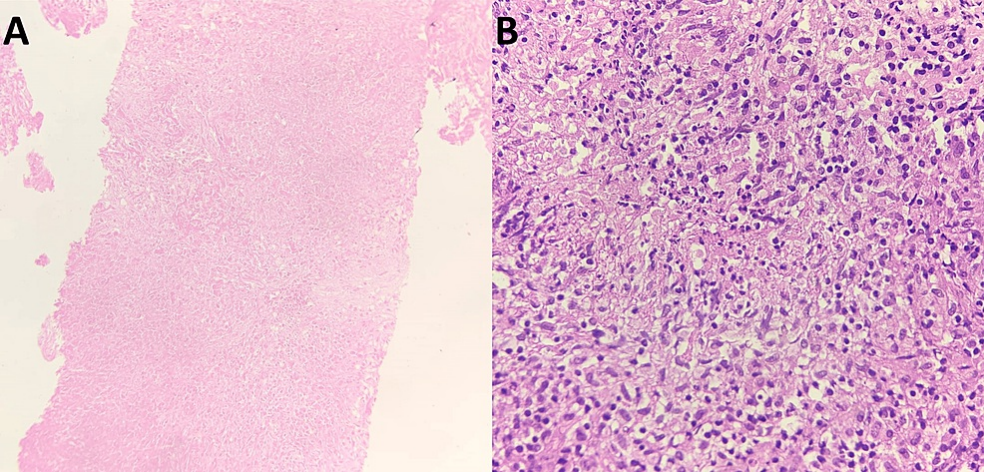
Haematoxylin and eosin (H&E) stained sections from the CT-guided biopsy. Pane A: Fragments of tissue which are predominantly necrotic (scanning magnification). Pane B: Ill-defined granulomata with necrotic foci interspersed with inflammatory cells (400x).

A 65-year-old female homemaker, with a history of type 2 diabetes mellitus, hypertension, hypothyroidism, and bronchial asthma, presented to the general medicine clinic with concerns of generalised fatigue, night sweats, and loss of appetite with significant loss of weight of around 12 kg over the past six months. On initial evaluation, she was noted to have an apathetic affect, a poorly nourished habitus, and pallor. The remainder of the physical examination was unremarkable, and the patient did not have lung signs. A routine evaluation revealed microcytic hypochromic anaemia with thrombocytosis, iron deficiency on iron studies, and albumin:globulin ratio reversal with otherwise normal renal and liver function tests (Table 1). Electrocardiogram, chest X-ray, and 2D-transthoracic echocardiogram were normal. Blood, sputum, and urine cultures yielded no growth. Two samples of stool were negative for occult blood. Anti-nuclear autoantibody (ANA) testing was negative. In view of age, significant weight loss, anaemia, and albumin:globulin ratio reversal, differential diagnoses included malignancy, chronic inflammatory disease, and chronic infection (such as tuberculosis). Hence, positron-emission tomographic computerised tomography (PET-CT) was done. This revealed a suspicious consolidating lesion in the right lung with bilateral pulmonary nodules and locoregional lymphadenopathy (Figure 1). A CT-guided biopsy of the right lung lesion was done, and histopathological examination showed epithelioid granulomata with necrosis (Figure 2). At this time, differential diagnoses considered were pulmonary tuberculosis, fungal pneumonia, and malignancy. As pulmonary tuberculosis is endemic to India, a clinical diagnosis of pulmonary tuberculosis was made, and empirical anti-tuberculous therapy was initiated.

**Table 1:** Basic laboratory workup at initial presentation. MCV, mean corpuscular volume; WBC, white blood cell count (leukocytes); ESR, erythrocyte sedimentation rate; HbA1c, glycated haemoglobin; BUN, blood urea nitrogen; TIBC, total iron binding capacity; TSH, thyroid stimulating hormone Reference ranges and units as applicable included with each parameter.

Haemogram	Patient Value	Renal Function Tests	Patient Value	Liver Function Tests	Patient Value	Other Tests	Patient Value
Haemoglobin (g/dL, 12-15)	8.8	BUN (mg/dL, 7.9-20.1)	10	AST (IU/L, <35)	12	Peripheral Smear	Microcytic hypochromic anaemia with thrombocytosis
MCV (fL, 83-101)	74.4	Creatinine (mg/dL, 0.6-1.2)	0.6	ALT (IU/L, <35)	9	Iron (μg/dL, 70-180)	<10
WBC (×10^3^/μL, 4-11)	11.7	Sodium (mmol/L, 136-146)	132	ALP (IU/L, 30-120)	88	TIBC (μg/dL, 240-450)	121
Platelets (×10^3^/μL, 150-450)	845	Potassium (mmol/L, 3.5-5.1)	4.6	Albumin (g/dL, 3.5-5.2)	3.4	Transferrin Saturation (%, >16)	8.6%
ESR (mm/hour, <37.5 for age)	140	Chloride (mmol/L, 101-109)	91	Globulin (g/dL, 2-3.5)	4.6	Ferritin (ng/mL, 13-150)	986
HbA1c (%, <5.6)	6.9	Bicarbonate (mmol/L, 21-27)	28	Total Bilirubin (mg/dL, 0.3-1.2)	0.32	TSH (μIU/mL, 0.27-4.2)	1.75

**Figure 3:**
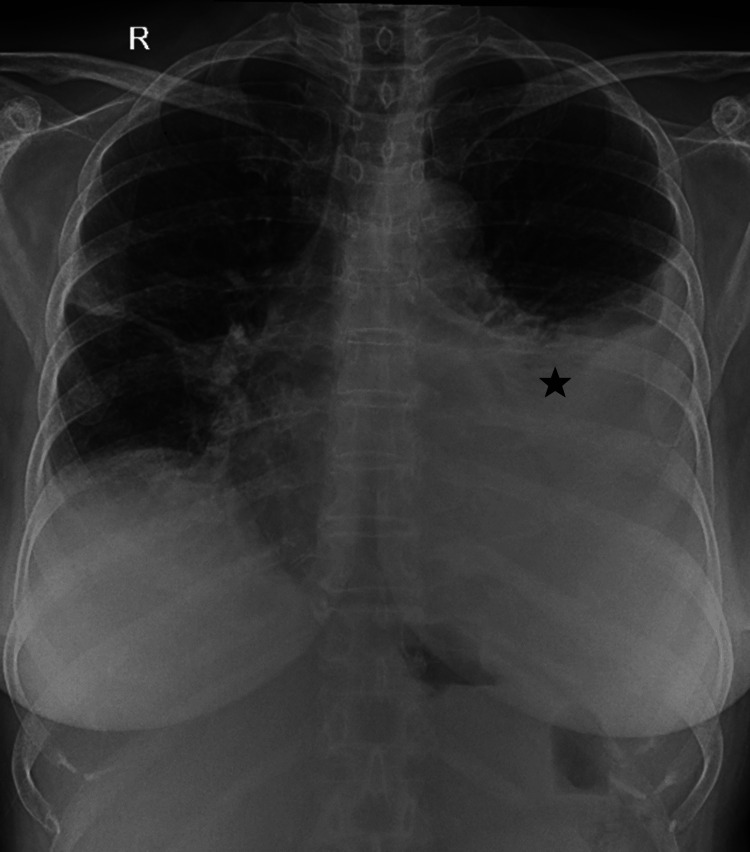
Chest x-ray revealing new-onset left pleural effusion (black star).

At follow-up three weeks later, patient continued to have fever spikes and poor appetite. Repeat chest X-ray showed lung opacities. Fungal stain of the CT-guided lung biopsy specimen revealed the presence of septate hyphae at acute angles, suggestive of pulmonary aspergillosis. Serum galactomannan was sent, which was 0.6 (normal <0.5 enzyme-linked immunosorbent assay (ELISA) units), considered as positive. Hence, patient was given antifungal therapy with parenteral voriconazole for two weeks. During this admission, haemoglobin was noted to drop further to 6.2 g/dL, for which work-up for blood loss was done. Upper gastrointestinal endoscopy, colonoscopy, and stool samples were unremarkable. The patient was transfused with one unit of packed red blood cells, following which haemoglobin rose to 7.6 g/dL. Repeat chest X-ray showed a unilateral left pleural effusion (Figure 3). The effusion was aspirated, and biochemical analysis revealed an exudative pattern with cell count 1,177 cells/μL of which 64% were mononuclear cells and 36% were polymorphonuclear cells, sugar of 182 mg/dL, adenosine deaminase of 23.2 IU/L (normal <33 IU/L), sterile cultures, negative acid-fast stain, and negative nucleic acid amplification testing (NAAT) for Mycobacterium tuberculosis. The patient was discharged with oral voriconazole maintenance therapy.

**Figure 4:**
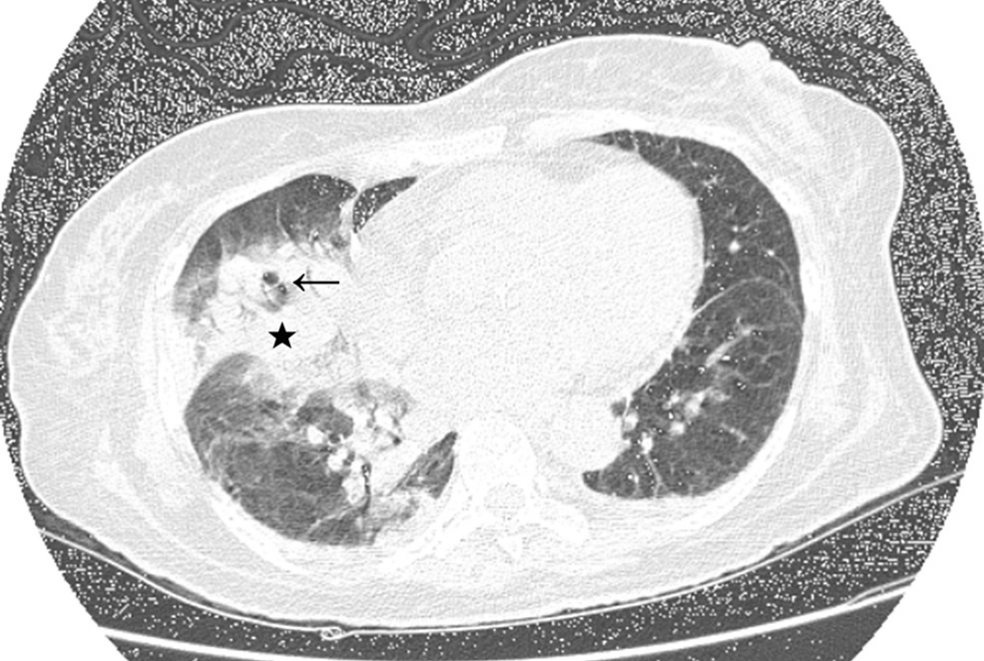
High-resolution CT of the thorax showing an increase in the size of the previous consolidation (black star) and presence of air bronchograms (black arrow).

**Figure 5:**
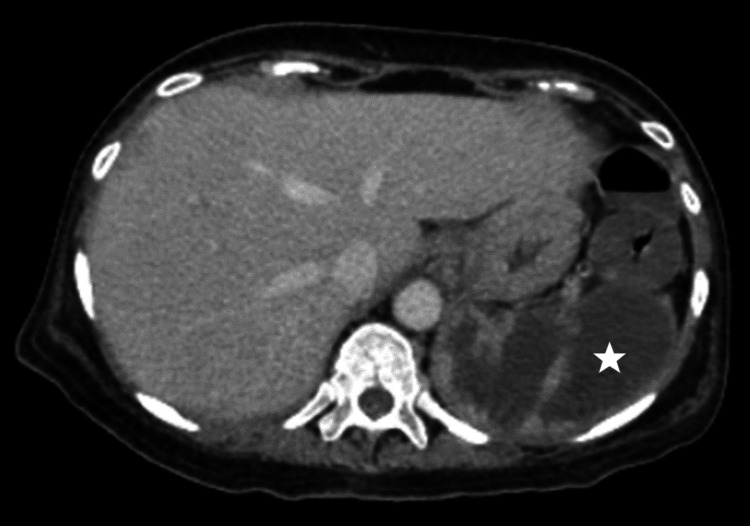
CT abdomen revealing loculated splenic collection (white star).

However, the patient presented one week after discharge to the emergency department in view of severe breathlessness at rest, chest pain and abdominal pain. Chest X-ray revealed bilateral infiltrates and perihilar shadowing suggestive of pulmonary oedema. New-onset electrocardiographic changes of T-wave inversions in inferior leads were noted; serum troponin I was 0.12 mg/dL (normal <0.02 ng/mL) and B-type natriuretic peptide was 1,150 (normal <100 pg/mL). Transthoracic echocardiography revealed new inferior wall motion abnormalities. The patient was admitted to the intensive care unit. In view of non-ST segment elevation myocardial infarction with pulmonary oedema, she was initiated on dual antiplatelets, a statin, anticoagulation, diuretics, and non-invasive ventilation. Ultrasound of the abdomen revealed a splenic collection of size 6.1×3.5×3.6 cm and volume 40 mL with free floating debris and no internal vascularity. High-resolution computerised tomography of the thorax revealed multiple areas of peribronchovascular consolidation across lung fields and significant interval increase in the size of the cavitatory lesions compared to previous PET-CT, as well as a prominent soft tissue density of 2×1.7 cm size in the right lung (Figure 4). Contrast-enhanced CT abdominal screening confirmed abdominal ultrasound findings (Figure 5). Ultrasound-guided aspiration of the splenic abscess yielded dark maroon fluid with neutrophil-predominant leukocytosis. Gram stain, acid-fast stain (for mycobacteria and Nocardia), and fungal stain were negative in this splenic fluid.

In view of splenic infarct/abscess and pulmonary nodules, the possibility of infective endocarditis was raised. Serial blood cultures (four samples) were sent, and the patient was started on empiric broad-spectrum antibiotics with intravenous piperacillin-tazobactam. Transoesophageal echocardiogram revealed a 7.3×6.3 cm vegetation at the base of the mitral valve with scalloping, mild mitral regurgitation, and no signs of valvular abscess. Blood cultures yielded no growth. In view of the lack of improvement in antibiotics and the presence of vegetation, systemic fungal disease was considered. Repeat serum galactomannan was 0.8 (normal <0.5 ELISA units). In the context of drug interaction involving voriconazole and rifampicin, serum voriconazole trough level was sent, with a value of 0.01 μg/mL (therapeutic trough range 1-6 μg/mL); hence, antifungal therapy was escalated to liposomal amphotericin B as rifampicin being an enzyme inducer caused subtherapeutic voriconazole trough levels. At this time, the patient developed a third-degree atrioventricular block for which temporary transvenous pacing was initiated.

**Figure 6:**
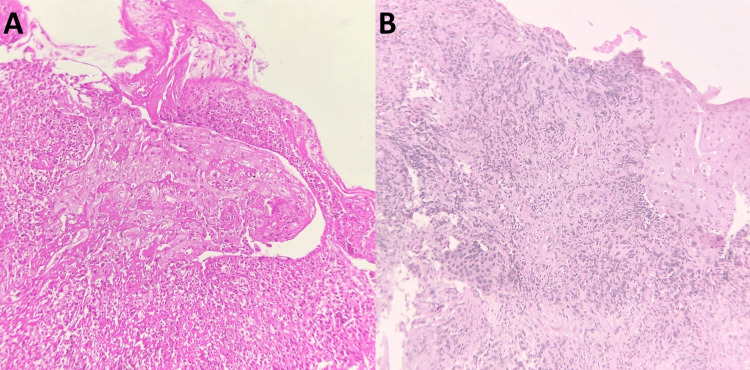
H&E stained sections from the bronchoscopic biopsy. Pane A: Stratified squamous epithelium with granulation tissue, capillaries with fibrinoid necrosis, and extravasated red blood cells (100x). Pane B: Stratified squamous epithelium with vessels showing focal fibrin deposits and neutrophils with karyorrhectic debris, representing leukocytoclastic vasculitis (100x).

Bronchoscopy revealed multiple nodular lesions in the larynx and trachea. Direct nasal endoscopy revealed the presence of similar nodular lesions in the nasopharynx. Biopsies from bronchoscopy and nasal endoscopy revealed leukocytoclastic vasculitis (Figure 6), with negative fungal staining and culture. Acid-fast stain, NAAT for TB, and bacterial and fungal cultures from the bronchial wash sample were negative. Antituberculous therapy was discontinued. The possibility of GPA was considered; a positive anti-proteinase 3 c-ANCA titre of 76.66 ELISA units (normal <11 ELISA units) was noted. Hence, a diagnosis of GPA with pulmonary, cardiac involvement (marantic endocarditis and conduction block), and splenic infarction was established. Repeat transoesophageal echocardiography showed no significant change in the size of the vegetations. In view of no other identifiable aetiology for the patient’s valvular vegetations and heart block, these were attributed to GPA.

At this time, a reduction in urine output and an increased creatinine (1.6 mg/dL) reflective of acute kidney injury were noted. A multidisciplinary team discussion was held, and the decision was made to induce immunosuppression. Initially, methylprednisolone pulse therapy (500 mg IV once daily) was given for five days, following which a dexamethasone maintenance dose was initiated. During the steroid pulsing period, a transient improvement in pulmonary function with reduced NIV requirement was noted; however, pulmonary function worsened again on the fourth day of steroid therapy. Worsening oxygenation and decline in sensorium led to elective endotracheal intubation and invasive ventilation; non-contrast neuroimaging and electroencephalography were unremarkable. A new-onset patch was noted on a repeat chest X-ray. In view of nosocomial infection with a vasopressor requirement, the patient was initiated on meropenem (2 g IV twice daily, as per renal adjustment) and teicoplanin (400 mg IV every other day, as per renal adjustment), and amphotericin B was continued. On multidisciplinary discussion, it was decided to attempt immunosuppression with rituximab. After initiation of rituximab (500 mg IV once weekly), a reduction in ventilatory pressure and oxygen requirement, improvement in sensorium, and mild improvement in urine output were noted. Two doses of rituximab were given. However, the patient developed recurrent fevers. Carbapenem-resistant Acinetobacter baumannii (CRAB) was isolated from the blood, and meropenem was escalated to polymyxin B (1.5 million units as loading dose, followed by 750,000 units twice daily). Rituximab was withheld with the intent to restart immunosuppression after improvement of the patient's general condition. Her fever improved thereafter. Kidney function and urine output did not improve, however. The patient required prolonged artificial ventilation and hence was tracheostomised.

Repeat chest X-ray revealed a new right-sided pleural effusion, which, on fluid analysis, was found to be transudative with cultures yielding no growth. In view of the recurrence of effusion despite multiple aspirations as well as the recurrence of fever, an intercostal drain was placed, and the fluid was sent for repeat analysis. These repeat pleural fluid cultures were exudative with polymorph-predominant leukocytosis and yielded significant growth of Elizabethkingia anophelis susceptible to co-trimoxazole and clindamycin. Co-trimoxazole was initiated (160/800 mg IV thrice daily).

Serial drop in platelet values was noted, decreasing from 25×10^3^ to 3×10^3^ cells/μL. Platelets were transfused. Pressor support requirement persisted. The patient had a cardiac arrest and, despite best resuscitative measures, could not be revived. In her last admission, the length of the patient's hospital stay was approximately three months.

## Discussion

GPA is a rare disease. Its incidence is region-dependent (around 2-10 cases per million people on average worldwide), and the disease primarily affects middle-aged people of European descent [2]. In India, most cases come from North India, with very few South Indian cases reported in the literature [3,4]. The presentation is often variable; any organ can be affected. The characteristic triad of organs most often involved are the upper respiratory tract (around 88%), the lower respiratory tract (75-95%), and the kidneys (77-95%) [5]. Ophthalmological involvement (40-45%) is primarily due to sino-orbital disease, although uveitis, scleritis, and rarely chorioretinitis can occur; cutaneous involvement (30-50%) may occur; and neurological involvement (15-30%), primarily in the forms of mononeuritis multiplex or cranial nerve entrapment due to sino-orbital disease, is also seen [2,5-7].

Cardiac involvement in GPA is heterogeneous in presentation and incidence. Though pericarditis and subclinical arrhythmias have been reported in up to 44% of cases, frank coronary artery disease seems to be much rarer (0.3-6%), and severe conduction defects and valvular involvement are rarer still (0.03-6%). Valvular disease in GPA presents commonly as stenotic or regurgitant lesions of the mitral or aortic valves, rarely with sterile vegetations [8,9]. Marantic endocarditis with conduction block, as seen in our patient, is an extremely rare presentation in GPA. To the best of our knowledge, cases of cardiac manifestations in the literature to date report valvular vegetations or conduction block, but not both together, making this the first case of synchronous presentation of endocarditis and conduction block secondary to GPA. Splenic infarction in our patient is likely a systemic embolic manifestation secondary to sterile endocarditis. Though previously considered to be a rare complication of the sterile endocarditis of vasculitic diseases, recent literature suggests that its prevalence may be underestimated [10].

Initially, GPA was a less likely differential during our clinical decision-making process, especially in the presence of histologically demonstrated fungal hyphae in the lower respiratory tract biopsy, as well as the positive serum galactomannan (which is generally above 75% specific for invasive aspergillosis [11]). Invasive *Aspergillus* pneumonia in a known asthmatic with secondary complications of fungal endocarditis, especially in the face of subtherapeutic levels of voriconazole therapy (likely secondary to concomitant administration of rifampicin [12,13]), despite repeated negative blood cultures, led to our decision to initiate of amphotericin B in this patient. Hence, it is prudent to check for drug interactions prior to the initiation of antifungal therapy when a patient is on enzyme inhibitors such as rifampicin. Infective endocarditis with ANCA positivity has been reported in the literature [14,15]. The presence of nodules in the upper respiratory tract, nonresolution of vegetations on broad-spectrum antibiotic and antifungal therapy, and persistently negative culture reports pointed towards small-vessel vasculitis as a more likely diagnosis. Improvement of the patient on initiation of immunosuppression supports the diagnosis of GPA.

This case demonstrates the difficulty in discriminating between the common presentations of common diseases (tuberculosis and invasive aspergillosis) and rare presentations of rare diseases (cardiac manifestations of GPA). With the advent of immunosuppressive therapy, 80% of patients diagnosed with GPA achieve remission to varying degrees based on the severity of the disease at presentation. Though these therapies can be lifesaving, morbidity and mortality secondary to immunosuppressive therapy also remain important obstacles to the achievement and maintenance of remission. Rituximab was chosen for our patient due to demonstrated noninferiority compared to cyclophosphamide in the literature [16], as well as the relatively less harmful adverse event profile. Even still, irreversible organ damage can occur consequent both to the disease and the treatment.

## Conclusions

Unresolving lung shadows warrant evaluation for fungal aetiology and noninfective causes. Our case report highlights the rarest cardiac presentation of sterile endocarditis, a conduction block with embolic manifestation of splenic infarction in a patient who had unresolved lung nodules and was later diagnosed as GPA. It is prudent to always check for drug interactions, especially when the patient is on enzyme inducers (such as rifampicin), as this may lead to subtherapeutic levels of co-prescribed medications (such as voriconazole).
